# Dental anxiety and potentially traumatic events: a cross-sectional study based on the Tromsø Study—Tromsø 7

**DOI:** 10.1186/s12903-021-01968-4

**Published:** 2021-11-23

**Authors:** Hege Nermo, Tiril Willumsen, Kamilla Rognmo, Jens C. Thimm, Catharina Elisabeth Arfwedson Wang, Jan-Are Kolset Johnsen

**Affiliations:** 1 The Public Dental Health Service Competence Center of Northern Norway, Tromsø, Norway; 2 Department of Clinical Dentistry, Faculty of Health Sciences, UiT The Arctic University of Norway, 9037 Tromsø, Norway; 3 Department of Pediatric Dentistry and Behavioural Science, University of Oslo, Oslo, Norway; 4 Department of Psychology, UiT The Arctic University of Norway, Tromsø, Norway; 5 Centre for Crisis Psychology, Faculty of Psychology, University of Bergen, Bergen, Norway; 6 Department of Clinical Dentistry, Faculty of Health Sciences, UiT The Arctic University of Norway, Tromsø, Norway

**Keywords:** Dental anxiety, Psychological distress, Psychological trauma, Sexual trauma, Oral health, Public health dentistry

## Abstract

**Objective:**

The objectives of the study were to describe the prevalence of dental anxiety and the possible associations between dental anxiety and potentially traumatic events in an adult population.

**Method:**

The study is based on cross-sectional questionnaire data from the 7th wave of the Tromsø Study, a study of the adult general population in the municipality of Tromsø carried out in 2015–2016. The Modified Dental Anxiety Scale was used to measure dental anxiety across potentially traumatic events, oral health, dental attendance (avoidance) and current mental health symptoms (Hopkins Symptom Checklist). Individuals with high and low dental anxiety scores were compared to investigate differences in the distribution of potentially traumatic events, current mental health symptoms, avoidance, sex and oral health, and hierarchical multivariable regression was used to study the influence of traumatic events on dental anxiety.

**Results:**

High dental anxiety was reported by 2.9% of the sample and was most prevalent among females and in the youngest age groups. Individuals with high dental anxiety reported more current mental health symptoms, and they were more likely to report poorer oral health and more irregular dental visits compared to individuals with no or lower dental anxiety scores. Concerning traumatic events, the reporting of painful or frightening dental treatment showed the biggest difference between those with high dental anxiety and low dental anxiety scores (a moderate effect). The hierarchical regression model indicated that reporting sexual abuse, traumatic medical treatment in hospital and childhood neglect significantly predicted dental anxiety in the step they were entered in, but only sexual abuse remained a significant individual contributor after controlling for current mental health symptoms.

**Conclusions:**

The prevalence of high dental anxiety was lower than expected (2.9%), but dentally anxious individuals expressed a high burden of mental health symptoms, poor oral health and the avoidance of dental care. The regression analysis indicated that experiences with sexual abuse could affect dental anxiety levels in the absence of generalised symptoms of anxiety and depression.

## Background

### Dental anxiety: prevalence, severity, aetiology

Individuals with high dental anxiety are often unable to utilise standard dental health services [1, 2]. Over time, a lack of adequate dental treatment results in more missing teeth and fewer filled and sound surfaces [3, 4]. The experience of high dental anxiety extends beyond the aversive emotional response that interferes with dental treatment; it may include flashbacks, nightmares, intrusive images, difficulties sleeping, irritability and impaired concentration, even if dental treatment is not imminent [5, 6]. Consequently, high dental anxiety often leads to reduced oral health-related quality of life [7–10] and has demonstrated comorbidity with generalised anxiety and depression disorders [11, 12]. The prevalence of high dental anxiety among adults is reported to be between 3.9% and 15.5% [9, 11, 13–20], depending on the measurements used and cut-off levels. Dental anxiety levels are reported to be higher among women and in younger age groups [13].

Conditioning theories, focusing on the development of a generalised fear response in the dental setting from previous painful and traumatic dental treatments [21, 22], are generally accepted as central to the acquisition of high dental anxiety but are an inadequate explanation in many respects [23, 24]. The experience and processing of a potentially traumatic or aversive event in a dental setting depend on many factors, including life history [25]. Nonetheless, pain is an important factor in conditioning experiences for developing and maintaining dental anxiety [26–28].

### Potentially traumatic events

Potentially traumatic events (PTE) are highly stressful and threatening events, such as exposure to violence, sexual abuse or neglect in childhood. Several studies have pointed to the negative impact of adverse childhood experiences on multiple health outcomes [29, 30], and similar associations with the development of oral disease may also be expected [31]. The brain reacts differently to traumatic events depending on sex, age, the possession of particular genotypes, personality characteristics, coping strategies, the nature of the trauma and the relationship to the perpetrator [22, 32]. In other words, the emotional load from aversive life events and the nature and timing are important in terms of how individuals respond and move forward after such events. Individuals with high dental anxiety have shown trauma-related symptoms similar to those of people suffering from posttraumatic stress disorder (PTSD) [5, 33]. Traumatic life events inflicted by others, like sexual or physical assault, are reported by 10–30% of the general population [34, 35]. These traumatic events, in particular, impact interpersonal relationships and can have severe consequences for the mental health and interpersonal functioning of the individuals that are affected [36].

### PTEs and dental anxiety

There are limited population studies concerning dental anxiety and PTEs outside the dental setting, and the evidence regarding whether or not there is an association in the general population is contradictory [5, 22, 37, 38]. However, studies on selected subgroups of the population indicate that victims of torture and violence and sexually abused women have a higher prevalence of dental anxiety than the general population [5, 39, 40]. We are not aware of any specific studies on male survivors of sexual abuse and dental anxiety but have no reason to believe that this association is different between the sexes. Trauma symptomology in survivors of childhood abuse exists independent of the sex of both the victim and the perpetrator [41]. Mental health symptoms are a measure of clinical importance concerning the long-term effects of PTEs and dental anxiety. However, to our knowledge, they have not been considered when examining the association between traumatic events and dental anxiety.

There are several hypotheses as to how traumas outside the dental clinic can influence dental anxiety. Situations, feelings and other cues from a traumatic experience in the past might be generalised to the dental treatment situation, following a broader conditioning pattern through traumatic coupling [42]. A dental treatment situation is intimate and can leave patients with a sense of a lack of control and helplessness, which could evoke reactions related to the past traumatic experience [5, 40]. A history of abuse and neglect is associated with difficulties in interpersonal relationships, the increased likelihood of depression and anxiety disorders and substance abuse [43, 44]. This burden might increase such patients' vulnerability to developing dental anxiety. It is possible to hypothesise that general anxiety disorders might be extended to include anxiety towards dental treatment or that potentially negative or painful dental experiences have a larger impact when mental health is compromised [45]. In other words, the development of dental anxiety could be driven primarily by the mental health challenges that follow traumatic experiences rather than the traumatic experiences themselves. It is a reliable finding that psychiatric disorders are associated with dental anxiety and that symptoms of depression and anxiety are predictive of dental anxiety over time [46]. Hence, symptoms of depression and anxiety could intermediate the association between traumatic events and dental anxiety. Since a dental treatment situation is arguably a highly relational, intimate and interpersonal circumstance [42], it comes as no surprise that dental experiences are inevitably coloured by the interpersonal relationship between the dental caregiver and the recipient [47–50].

Regarding sexual abuse, there is evidence indicating a more direct conditioning pattern. For instance, sexually abused women that had experienced oral penetration as part of the abuse reported higher dental fear than sexually abused women without experiences of oral penetration [40]. Studies have indicated that the response to triggers is overwhelming to the point of blurring previous memories of abuse and the current experiences in the dental chair [51]. Nonetheless, there is limited research on how traumatic events involving interpersonal relationships influence dental anxiety in the general population. Moreover, current mental health symptoms have not yet, to our knowledge, been considered in research on dental anxiety and traumatic events.

The objectives of the present study were to investigate the associations between dental anxiety, oral health and mental health symptoms, as well as the potential influence of PTEs on dental anxiety in the general population. The specific aims were as follows:

1. To describe the prevalence of high dental anxiety within a large population-based study of individuals aged 40 and above in Northern Norway (the Tromsø 7 study).
2. To analyse the influence of PTEs on dental anxiety when controlling for sex, age, oral health, dental attendance (avoidance) and current mental health symptoms.
3. To analyse the mediating effect of current mental health symptoms on the relationship between PTEs and dental anxiety.

## Method

### Study population and design: the Tromsø Study

The Tromsø Study [52] is a repeated population health survey. This study used data collected from the 7th survey of the Tromsø Study, in which all adults registered in Tromsø municipality aged 40 or above were invited to participate (32,591 in total). The registration and examination of participants took place from 2015 to 2016. In total, 21,083 residents (10,009 males and 11,074 females) took part in the study, which resulted in a participation rate of 65%. The participants answered questionnaires about several different health topics and received a physical health examination. In the present study, only the questionnaire data were used. Detailed information on the questionnaires and study data is provided through the Tromsø Study web resource [https://uit.no/research/tromsostudy] [53]. All participants gave written consent. The Regional Committee of Medical and Health Research Ethics (reference number 2018/1352/REK nord) approved the present study in July 2018.

## Measures

### Age, ethnicity, household income and education

Age was registered based on year of birth, and it was moderately skewed in the current population. Based on this skewness in birth years and our focus on differences over age span, the choice was made to reduce age into eight age categories, spanning five years in each category (40–44, 45–49, etc.). Due to few participants above the age of 75 years, these were included in one category. The ordinal age variable was used as a continuous predictor in the analyses due to visual inspection and low skewness and kurtosis values.

Ethnicity was registered by asking “What do you consider yourself to be?” with the options “Norwegian”, “Sami”, “Kven/Finnish” or “other”. It was possible to choose more than one option.

Participants were to indicate last years household income before taxes by choosing one out of eight categories ranging from “less than 150.000NOK” to “more than 1.000.000NOK”.

The highest level of education was registered in four categories, "10 years of primary education", "three years of upper-secondary education", "less than four years in college or university", and "more than four years in college or university".

### Dental anxiety

Dental anxiety was measured using the modified dental anxiety scale (MDAS) [14]. The MDAS is a five-item scale, with response options ranging from "not anxious" to "extremely anxious", yielding a score from 5–25. This variable was dichotomised in the chi-square analysis with a recommended cut-off value of 19 [15]. The MDAS had a Cronbach's alpha of 0.93 (N = 20,197) and a mean inter-item correlation of 0.74.

### Current mental health symptoms

The current mental health symptoms of the study participants were measured using the 10-item Hopkins Symptom Checklist (HSCL-10), validated for use in epidemiological studies as an indicator of symptoms of depression and anxiety during the last two weeks [54]. Each question is answered based on four-point Likert scale, ranging from 1 ("not bothered") to 4 ("very bothered"). Mean item scores were calculated, and the variable was used as a continuous scale in the regression analyses and dichotomised in the bivariate analysis above and below the score of 1.85, indicating symptoms of mental illness [54]. The Cronbach's alpha of the HSCL-10 was 0.876 (N = 19,824), and it had a mean inter-item correlation of 0.41.

### Oral health

Oral health was assessed with a single question: "How do you consider your oral health to be?" The participants selected one out of five response options to this question: "excellent", "good", "neither good nor bad", "bad", or "very bad". The scores were dichotomised in the bivariate analyses. Good (0) included the responses "excellent" and "very good", while bad (1) comprised "neither good nor bad", "bad" and "very bad". In the hierarchical multivariable regression, the Likert format response was used as a continuous variable, assuming that the distances between the answers were equal and meaningful. Both visual inspection and the low skewness and kurtosis values indicated that these items could be treated as numerical. Also, the regression was run with the dichotomised variable, which yielded the same result.

### Avoidance

Dental attendance patterns were measured by the item, "Do you go to the dentist regularly?", where participants could indicate their attendance patterns by choosing one of six alternatives: "at least once a year", "every year", "every second year", "less than once every two years", "only for acute problems" and "never". This variable was dichotomised to indicate the avoidance of dental treatment, in which yes (0) included the response alternatives "at least once a year", "every year", "every second year" and" less than once every two years", and no (1) included the response alternatives "only for acute problems" and "never".

### Potentially traumatic events (PTEs)

A list of eleven PTEs was included in the Tromsø 7 Study with four response options (no; yes, before the age of 18; yes, after the age of 18; yes, in the last year). The list of PTEs was developed by the Clinical Psychology Research Group at UiT, The Arctic University of Norway, with the aim of assessing a broad range of PTEs while keeping the number of items low due to restrictions imposed by the Tromsø 7 study. The selection of items was based on a review of existing measures and consultation of experts in the field. Many items overlap with other PTE items used elsewhere [5, 55, 56]. In this study, events that had the potential to affect interpersonal relationships were included: painful or threatening dental treatment, painful or threatening medical treatment in hospital, childhood neglect, violence, sexual assault, bullying or witnessing someone close being a victim of violence or sexual assault. The answers were dichotomised into 0 = no and 1 = yes to avoid deflated effect sizes after assuring that the direction of the effects was the same regardless of the timing of the event.

## Analyses

All the analyses were performed using IBM Statistical Package for the Social Sciences (SPSS) Statistics version 26 (IBM Corp., Armonk, NY, USA). Chi-squared tests for independence were used to explore the relationship between the dichotomised MDAS and the independent variables used in the analysis. Hierarchical multivariable regression was used to assess the ability of selected PTEs (sexual abuse, violence, bullying, witness, childhood neglect, painful or threatening medical treatment at the hospital) to predict levels of dental anxiety when controlling for sex, age, self-assessed oral health, the avoidance of dental treatment and painful or threatening dental treatment. The demographic control variables, sex and age, were entered in the first step, followed by the known predictors of dental anxiety from the dental setting in the second step and all the traumatic life events listed above in the third step. HSCL-10 was entered in the final step to assess how it influenced the model and the effect of the predictors on dental anxiety. Preliminary analyses on the assumptions for linear regression indicated issues with the normal distribution. When inspecting the outliers, all of them reported a higher dental anxiety level than predicted by the model; however, they are a natural part of the population and were therefore not removed or transformed for the current analysis. Hence, the distribution of the test statistics was empirically derived through bootstrapping with 1000 bootstrap samples. Simple mediation analyses were performed using the PROCESS macro in SPSS [57] with 6000 bootstrap samples. Analysing the dataset with regard to missing values disclosed that 2.4% of all the values were missing, 9.6% of all the subjects had incomplete data, and there were missing values in all but two variables (sex and age). The items that made up our variable for measuring mental health symptoms (HSCL-10) had the most missing data (6%), followed by the MDAS (4.2%), avoidance (2.3%) and oral health (2.0%). All the PTEs had the same number of missing values (1.1%). The Little's missing completely at random (MCAR) test was significant, which indicated that data were not missing completely at random. The continuous variables (HSCL-10, MDAS, and oral health) were compared with a version of themselves where the missing values were replaced by the series mean using a paired sample t-test to evaluate the impact of the missing data. However, since the standard error of the difference was zero, it was not possible to perform any of these analyses. A comparison with the original dataset and the dataset with imputation of missing data was also performed, using both regression and expectation–maximisation techniques. The differences between the variables in the two datasets were limited to deviations in the second decimal. Finally, a regression analysis was performed by replacing the missing values with multiple imputations, which yielded similar results as the original data analysis. The differences were limited to decimal levels in the unstandardised regression coefficient, and there were no differences in β when rounded up to the first decimal place. Based on these findings, the analyses were performed on the original data without any imputation with the pairwise exclusion of missing data.

## Results

Almost half of the participants in this study reported higher education levels, and 30% had more than four years of university education. Over 94% of the participants specified their nationality as Norwegian, 2.8% as Sami and 1.9% as Kven, and 4.2% felt they belonged to another ethnicity. Most of the participants fell in the second age group (45–49), which constituted 15.6% of the population under study. The age groups with the least participants were the oldest (70–74 and 75+), representing 8.1% and 8.2% of the population, respectively.

The mean sum score of the MDAS was 7.7 (SD = 3.8), with a median of 6 (IQR = 4.0). The prevalence of highly anxious (MDAS ≥ 19) participants in this study was 2.9%. The mean value of the MDAS among the highly anxious was 21.7 (SD = 2.2), and the median was 21 (IQR = 4.0). More than 40% of the population scored at the minimum (sum score 5), and 6.6% scored ≥ 15, which corresponds to the cDAS score of 12 [58]. Current psychological symptoms (mean score ≥ 1.85) were indicated by 8.7% of the participants (10.7% of the females compared to 6.5% of the males). The majority reported good oral health (54.9%), and females scored their oral health a little higher than males, with 58.9% having good or excellent oral health compared to 50.6% of the men. The avoidance of dental health services was reported by 10.2%, with fewer avoiders being observed among females (7.8%) than among males (12.8%). Nearly a quarter of the population under study (23.8%) had experienced one or more traumatic and/or painful dental experiences, with little difference between the sexes (females: 23.7%, males: 23.8%).

### Associations of dental anxiety with age, education, income and oral health

**Fig. 1:**
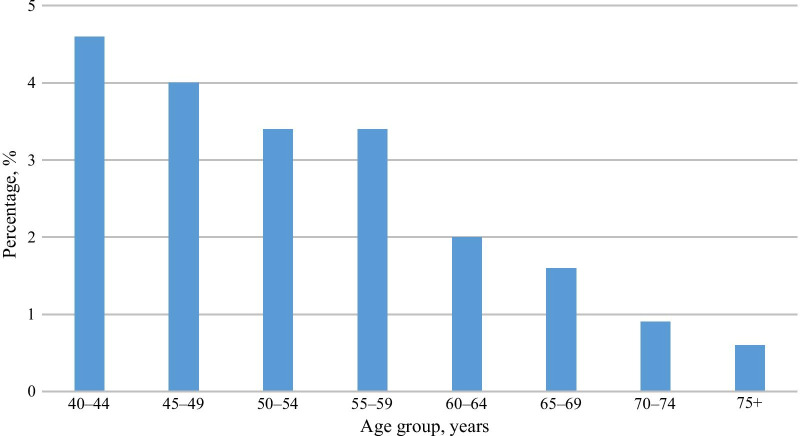
Prevalence of high dental anxiety (MDAS ≥ 19) by age group

Spearman’s correlation showed that dental anxiety scores significantly declined with age; *rs* (n = 20,195) =  − 0.25,* p* < 0.001, and comparing the youngest (MDAS; median = 7) and the oldest (MDAS; median = 5) groups with a Mann–Whitney U test revealed a medium to large effect; *U* = 3,476,217, *z* = 226.92, *p* < 0.001, *r* = 0.4. The proportions of highly anxious individuals were also significantly higher in the younger age groups (Fig. 1).

The dental anxiety scores varied inconsistently across different levels of education and household income; however, the proportion of individuals with MDAS ≥ 19 was significantly higher in groups with lower income and lower education (Table 1). We performed Chi-squared tests of independence to test these relationships. There was a small significant difference between high and low dental anxiety and level of educational, χ^2^ (3, N = 19,287) = 27.38, *p* < 0.01, Cramer’s V = 0.04. Similarly, there was a small significant difference between high and low dental anxiety and income χ^2^ (7, N = 19,430) = 56.36, *p* < 0.01, Cramer’s V = 0.05.

**Table 1:** Frequency breakdown and N size for respondents including MDAS total score means (SD) and percent ≥ 19

	N	%	Mean	Median	SD	% 19+
*Household income NOK*						
< 150.000	210	1.0	7.9	5.0	4.5	5.6
150.000–250.000	990	4.9	7.6	5.0	4.5	5.4
251.000–350.000	1439	7.1	7.7	6.0	4.2	4.2
351.000–450.000	1906	9.4	7.6	6.0	3.9	2.9
451.000–550.000	2312	11.5	7.8	6.0	3.9	2.9
551.000–750.000	3572	17.7	7.7	6.0	4.0	3.0
751.000–1.000.000	4741	23.6	7.9	7.0	3.8	3.0
> 1.000.000	5015	24.8	7.6	6.0	3.3	1.7
*Education*						
Primary^a^	4796	23.1	7.5	5.0	4.0	3.6
Upper secondary^b^	5756	27.8	7.8	6.0	4.0	3.4
Tertiary < 4 years^c^	4008	19.4	7.7	6.0	3.7	2.8
Tertiary > 4 years^d^	6145	29.7	7.7	7.0	3.4	2.1
*Ethnicity*						
Norwegian	19,835	94.1	7.6	6.0	3.8	2.9
Sami	580	2.8	7.9	7.0	4.0	3.2
Kven/Finnish	400	1.9	8.0	7.0	3.9	3.1
Other	884	4.2	8.0	7.0	3.8	2.9
*Oral health*^*e*^						
Excellent	3143	15.2	6.9	6.0	2.8	1.1
Good	8203	39.8	7.3	6.0	3.1	1.4
Neither good nor bad	7263	35.2	7.9	7.0	3.8	2.9
Bad	1497	7.2	9.5	8.0	5.4	10.4
Very bad	547	2.6	10.5	8.0	6.7	16.7

^a^Primary and lower secondary, up to 10 years of education. ^b^Minimum of 3 years. ^c^College or university less than 4 years. ^d^College or university 4 years or more. ^e^"How do you consider your oral health to be?"

As shown in Table 2, chi-squared tests for independence (with Yates' continuity correction) indicated a significant difference in individuals with high and low dental anxiety scores and oral health. Dentally anxious individuals were more inclined to report very bad oral health (71.7%) compared to individuals with lower dental anxiety scores (11.9%). Furthermore, among the dentally anxious, 39.9% never went to the dentist or only went for acute problems versus 9% among those with lower dental anxiety levels (Table 2). There were also significant associations between high or low dental anxiety scores between the sexes and current mental health symptoms (Table 2). No significant differences were found for dental anxiety between different ethnicities. When repeating this analysis separately for the sexes, anxious males reported significantly poorer oral health (90.2% reported very bad oral health) compared to anxious females (61.1% reported very bad oral health). More anxious (MDAS ≥ 19) females (69.6%) than anxious males (41.2%) reported going to the dentist regularly.

**Table 2:** The distribution of low and high dental anxiety in key variables

Variable	N	% Low dental anxiety	% High dental anxiety	χ^2a^	PHI^b^
*Sex*					
Female (0)	10,583	52	66.6	47.63*	−0.05
Male (1)	9614	48	33.4		
Total count	20,197	19,614	583		
*Current mental health symptoms*					
HSCL < 1.84	17,729	91.8	75.7	170.74*	0.09
HSCL ≥ 1.85	1686	8.2	24.3		
Total count	19,415	18,867	548		
*Avoidance*^*c*^					
Never, only for acute problems (0)	1984	9.0	39.9	602.69*	0.17
Goes regularly (1)	18,160	91	60.1		
Total count	20,144	19,565	579		
Oral health^d^					
Excellent (0)	3022	88.1	28.3	323.69*	0.31
Very bad (1)	484	11.9	71.7		
Total count	3506	3393	113		

Frequency of high (≥ 19) and low (< 19) dental anxiety scores (MDAS) according to sex, mental health symptoms, dental attendance (avoidance) and oral health

^a^Yates' correction for continuity; Chi-square test of independence

^b^Phi coefficient (effect size: 0.10 for small effect, 0.30 for medium effect, 0.50 for large effect [Cohen’s criteria 1988])

^c^Do you go to the dentist/experience dental care regularly? 0 = no, never or only for acute problems 1 = yes, often, yearly, every other year or less than once every two years

^d^Comparing high and low evaluations of one's own oral health

*p < 0.01

Chi-square tests of independence were used to test the proportion of traumatic experiences in the high and low dental anxiety groups and the strength of the associations between the two (Table 3). There were significant differences in dental anxiety for all the traumatic events, and the effect sizes were small and highest for traumatic and/or painful dental experiences (phi = 0.18).

**Table 3:** The proportion of reported PTEs in the high and low dental anxiety groups

Trauma	MDAS ≥ 19	MDAS < 19	χ^2a^	Phi^b^	OR	95% CI
Any trauma	82.3	51.4	212.23*	0.10	4.4	3.54–5.44
Dental trauma^c^	69.0	22.7	660.49*	0.18	7.6	6.32–9.04
Sexual abuse^d^	20.8	9.2	85.21*	0.07	2.6	2.09–3.16
Violence^e^	26.4	15.2	53.60*	0.05	2.0	1.66–2.42
Bullying^f^	32.1	21.1	39.77*	0.05	1.8	1.48–2.11
Witness^g^	16.6	9.1	37.39*	0.04	2.0	1.60–2.50
Medical trauma^h^	20.6	10.6	57.38*	0.05	2.2	1.78–2.69
Failure of care^i^	13.0	6.7	34.58*	0.04	2.1	1.64–2.69

The proportion of reported PTEs in the high and low dental anxiety groups and the strength of the associations (odds ratios) between traumatic events and anxiety level

*p < 0.01

^a^Yates' correction for continuity; Chi-square tests of independence

^b^Phi coefficient [effect size: 0.10 for small effect, 0.30 for medium effect, 0.50 for large effect (Cohen’s criteria 1988)]

^c^Received painful or terrifying medical treatment at the dentist

^d^Been exposed to sexual abuse, i.e. sexual actions against your will

^e^Been exposed to violence (for example, hit, kicked, beaten, robbed or threatened with a firearm)

^f^Been called negative things, marginalised, threatened or bullied by schoolmates, fellow students or coworkers over a long period of time

^g^Witnessed someone close to you being exposed to violence or sexual abuse

^h^Received painful medical treatment when hospitalised due to sickness or serious injury

^i^Failure of care in childhood, i.e. not having received the necessities of food, clothing, protection and care/love from parents/caregivers

### Predicting dental anxiety from PTEs

The results of this analysis are given in Tables 4 and 5. The control variables entered in steps 1 and 2 in Table 5 explained most of the variance in dental anxiety (23.1%). In the final model, the only PTE entered in step 3 that remained a significant contributor to the model was sexual abuse; B = 0.220, SE = 0.086, β = 0.017, *p* = 0.011. All the control measures entered in the two first stages remained statistically significant (*p* < 0.001).

**Table 4:** Model summary of the hierarchical regression analysis with the MDAS as the dependent variable

	ΔF(*df*1,*df*2)	ΔR^2^
Model 1	646.68* (2,19012)	0.06
Model 2	1374.83* (3,19009)	0.17
Model 3	8.63* (6,19003)	< 0.01
Model 4	341.72* (1,19002)	0.01

*p < 0.01

**Table 5:** Hierarchical regression with the MDAS as the dependent variable

	B	SE B^I^	β	Sig.^i^	CI 95%^I^	
					Lower	Upper
*Step 1*						
Constant	9.58	0.07		< 0.01	9.45	9.72
Sex	− 1.08	0.05	− 0.14	< 0.01	− 1.18	− 0.97
Age	− 0.36	0.01	0.20	< 0.01	− 0.38	− 0.34
*Step 2*						
Constant	11.38	0.14		< 0.01	11.10	11.67
^a^Oral health	− 0.67	0.03	− 0.16	< 0.01	− 0.73	− 0.61
^b^Avoidance	1.91	0.12	0.15	< 0.01	1.67	2.15
^c^Pain dentist	2.55	0.07	0.30	< 0.01	2.4	2.67
*Step 3*						
Constant	11.20	0.15		< 0.01	10.91	11.49
^d^Neglect	0.23	0.11	0.02	0.04^II^	0.01	0.45
^e^Violence	0.01	0.08	0.00	0.88	− 0.15	0.17
^f^Sexual abuse	0.38	0.10	0.03	< 0.01	0.19	0.58
^g^Bullying	0.09	0.07	0.01	0.22	− 0.05	0.22
^h^Witness	0.09	0.10	0.01	0.39	− 0.12	0.28
^i^Hospital pain	0.21	0.09	0.02	0.03^II^	0.04	0.41
*Step 4*						
Constant	9.36	0.18		< 0.01	8.98	9.73
^j^HSCL-10	1.25	0.09	0.13	< 0.01	1.09	1.42

^I^Confidence intervals (CI) and standard errors (SE) are based on 1000 bias-corrected and accelerated bootstrap samples

^II^Lost their significant contribution in the last model (see figure on mediation)

^a^Self-reported oral health (Likert format)

^b^Avoidance: 0 = goes regularly to the dentist, 1 = never goes or only for acute dental pain

^c^Received painful or terrifying medical treatment at the dentist

^d^Failure of care in childhood, i.e. not having received the necessities of food, clothing, protection and care/love from parents/caregivers

^e^Been exposed to violence (for example, hit, kicked, beaten, robbed or threatened with a firearm)

^f^Been exposed to sexual abuse, i.e. sexual actions against your will

^g^Been called negative things, marginalised, threatened or bullied by schoolmates, fellow students or coworkers over a long period of time

^h^Witnessed someone close to you being exposed to violence or sexual abuse

^i^Received painful medical treatment when hospitalised due to sickness or serious injury

^j^Hopkins Symptom Checklist: current mental health symptoms

**Fig. 2:**
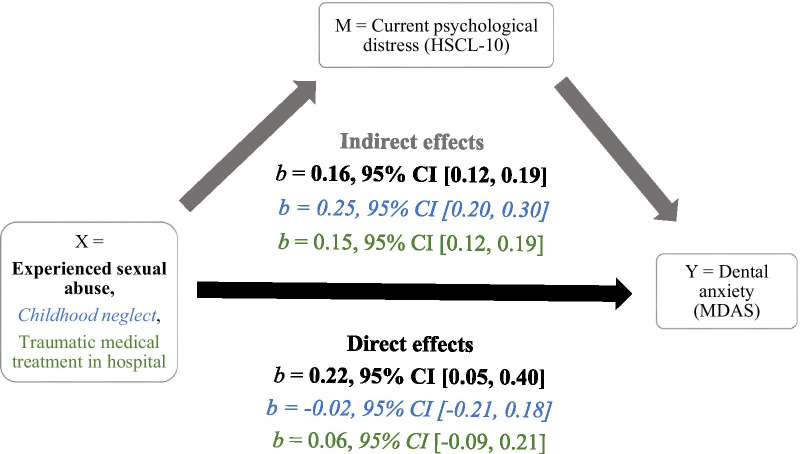
Mediation analyses. *Legend* Analyses of how the HSCL-10 mediates the effect of three potential traumatic life events on dental anxiety scores using the PROCESS macro in SPSS. The figure contains three analyses that are distinguished through both colour coding and the use of bold and italic text formatting. All events are mediated through the HSCL-10 (indirect effect), but only sexual abuse continues to have a significant direct effect on dental anxiety when accounting for the effect of mediation through the HSCL-10

As it is well known that experiencing traumatic events may increase the risk of mental health symptoms [59, 60], and that there is a relationship between mental health symptoms and dental anxiety [11, 61], the mediating effect of mental health symptoms was investigated in three separate simple mediation models. The predictor variables of sexual abuse, childhood neglect and traumatic medical treatment in the hospital were tested with the HSCL-10 as the mediator and the MDAS as the outcome (Fig. 2). All the other covariates from the regression model were included in these analyses. Mental health symptoms mediated the effect of the three PTEs investigated, but sexual abuse had a larger direct effect on dental anxiety than an indirect one, hence the continued significant effect in our hierarchical regression model. The effects of childhood neglect and traumatic medical treatment were fully mediated through current mental health symptoms (HSCL-10) (Fig. 2).

## Discussion

To our knowledge, this is the first study to investigate the association between dental anxiety and PTEs while considering mental health symptoms and self-reported oral health.

The present study investigated dental anxiety in the 7th wave of the Tromsø Study (> 40 years old), and in accordance with the specific aims, this population expressed a lower prevalence of dental anxiety than expected [9, 11, 13, 16, 62]; however, this study confirmed previously reported associations between dental anxiety and sex, age, dental attendance, previously traumatic or painful dental treatments and oral health [13, 16, 20, 63].

This study also found that the following PTEs outside the dental setting had a significant impact on our hierarchical regression model at the stage they were entered: sexual abuse, painful or scary medical treatment at the hospital and childhood neglect. Of these, only sexual abuse remained a significant predictor after controlling for current mental health symptoms. Furthermore, the mediation analysis provided evidence that current mental health symptoms seem to mediate the effect of traumatic or painful medical treatment in hospital, sexual abuse and neglect in childhood on dental anxiety. However, sexual abuse was only partially mediated.

The change in R-squared in step 3 in the hierarchical model (Table 4) is low. PTEs by themselves do not generate precise predictions of dental anxiety scores. While trauma history can be highly relevant in many clinical cases of severe dental anxiety, it does not account for the variance across all levels of dental anxiety in a general population. The items display a poor fit to the model, probably due to the systematic differences between the observations and the predicted values. The individual response depends on the severity of the event, the developmental level at the time of trauma, prior and subsequent life events, biological and social factors [64], none of which is accounted for in this model. An individual’s response to a PTE is as important as the event itself. Hence, the statistically significant associations are not negligible in the clinical understanding of highly dentally anxious individuals with a trauma history, despite the low R-squared value in the regression model.

The experiences of sexual abuse appear to impact dental anxiety in individuals irrespective of whether the same individuals are experiencing generalised mental health problems (depression, anxiety). This could point to sexual abuse experiences being somehow directly conditional on dental anxiety [42] and clarify the lack of conscious awareness of the link between sexual abuse and dental anxiety, which might be evident in some survivors of sexual abuse [40]. Evidence shows that many aspects of dental treatment can trigger memories and flashbacks from sexual abuse within the concept of trauma coupling [42, 50, 65]. Crossing lines of intimacy in the dental setting can be difficult in itself, but the oral cavity may have been violated directly, which could make the dental setting even more challenging [40, 66]. Remaining motionless, not having the ability to speak and not seeing what the dentist is doing may echo feelings of loss of control and being violated all over again [51, 65, 66]. The dental personnel's reassurances can also trigger memories since perpetrators often do this as well as a means of justifying their actions or controlling the situation. This might include telling victims to keep calm, that it will not hurt and that it will be over soon [67].

The regression analysis underlines the importance of traumatic dental treatment, oral health and the avoidance of dental treatment as predictors of dental anxiety. However, it is worth noting that most of the variance in dental anxiety is unaccounted for in this model, which points to the fact that there is more to explaining variance in dental anxiety than the factors included in the current model. One aspect that was not controlled for was personality factors, such as neuroticism. Neuroticism is considered to impact health in general [68] and dental anxiety specifically [12, 61]. Another factor not considered was oral health-related quality of life, which evidence shows is often impaired in dentally anxious individuals [69]. Oral health affected by dental anxiety and subsequent avoidance behaviour may affect the oral health-related quality of life and lead to social isolation and the presentation of symptoms related to depression and general anxiety [70, 71]. The socioeconomic variables (education and income) were left out of the regression analysis due to their lack of significant impact in the first trial model (not reported). However, it is known that socioeconomic status impacts oral health [72], and there is evidence that social class affects communication and trust in the patient-caregiver relationship [73]. Dental anxiety has a multifactorial aetiology; hence, effect sizes for the single variables are not expected to be high. Identifying these variables is important, but they must be considered part of a set of variables to understand the clinical variations in the population.

The avoidance of dental treatment is much more prevalent among anxious individuals compared to the rest of the population. Still, 60% of the dentally anxious participants reported going regularly to the dentist, and 89.8% of all the participants in this study reported a regular attendance pattern. Individuals with a dental phobia who chose not to participate in this study would probably be more inclined to use avoidance as a strategy than the phobic participants, which would influence the reported attendance pattern. Note that the reporting of regular dental treatment includes the option "less than once every two years", an alternative that could be considered to be more irregular.

There is some controversy in terms of performing mediation analyses on cross-sectional data since the order of occurrence of reported measures is unknown in an individual's life. The HSCL-10 measures current symptoms (the last two weeks), and it is reasonable to assume that PTEs most likely happened sometime previous to the last two weeks in these types of studies. Similarly, the MDAS is also a current measure, making it impossible to pinpoint the onset of dental anxiety in the past. Even though the order of events can be stipulated or hypothesised, causal effects cannot be ascertained, and the direction of correlation in the mediation analyses is probably, to some extent, bidirectional. Current mental health symptoms might affect reported PTEs [74], and high dental anxiety could influence current mental health symptoms. Nevertheless, there is a well-documented link between traumatic life events and mental health. It could be argued that some traumatic life events might affect dental anxiety as a result of causing increased vulnerability in an individual. That is, the psychological consequences of traumatic experiences might increase susceptibility to dental anxiety and poorer oral health.

There was a low prevalence of highly anxious participants in this study compared to previous investigations, as only 2.9% of the participants scored over the cut-off value of 19 for high dental anxiety. This could be interpreted as a result of a continuous decline in dental anxiety in the adult population [13] or reflect differences in the measures used to classify high dental anxiety. Still, the possibility of issues with the representativeness of individuals with high dental anxiety in the study population cannot be ruled out, especially considering that some of the participants were to undergo a thorough clinical dental examination. This could, to some extent, hinder recruitment among highly dentally anxious individuals due to the avoidance behaviour that characterises the phobic disorder [75]. This is the obvious pitfall of all population studies in the field. However, the questionnaire data used were collected independently of the dental examination, so the dentally anxious could participate in the study while declining the oral examination. Also, if considering the possible bias of representativeness, it can be inferred that the associations in all the bivariate analyses are supported by the existing literature, and including a larger portion of dentally anxious individuals would probably enhance rather than lower the effect sizes of the correlations observed.

Self-reported measures without any clinical markers are problematic and can give rise to bias. Mapping of past life trauma has uncertainties that affect the validity and reliability of PTEs. It could threaten the validity due to labelling and self-definition issues or reporting impediments tied to stigma and shame [76]. Memory deficits ranging from incomplete recall to psychogenic amnesia (dissociative amnesia, blocking out certain information) also threaten the validity and reliability of reporting PTEs. However, self-reports of abuse and neglect tend to be stable over time, and because the influences on memory serve mainly to inhibit recall or disclosure, it seems fair to conclude that reports confirming events should be given more weight than negative reports [77].

## Conclusion

The findings of this study show that survivors of sexual abuse can experience challenges in terms of receiving dental treatment regardless of whether their history of sexual abuse affects their current mental health status. Dental personnel should integrate this knowledge into their daily practices, recognise signs of sexual abuse, avoid re-traumatisation and refer patients to specialists in the field.

## Data Availability

Researchers affiliated with approved research institutions can apply for access to research of the Tromsø Study's material, available from The Tromsø Study [https://uit.no/research/tromsostudy]. All projects applying for data in the Tromsø study must have their own approval from the Regional ethics committee (REK).

## Abbreviations

PTE: Potentially traumatic event
MDAS: Modified dental anxiety scale
PTSD: Posttraumatic stress disorder
HSCL-10: Hopkins symptom checklist
SPSS: IBM statistical package for the social sciences
